# Supplementary material for: Evaluation of the PSMA-Binding Ligand 212Pb-NG001 in Multicellular Tumour Spheroid and Mouse Models of Prostate Cancer
Source: Int J Mol Sci. 2021 May 1;22(9):4815. doi: 10.3390/ijms22094815 (PMC8124365; doi:10.3390/ijms22094815)
Supplement: Supplementary file 1 [file ijms-22-04815-s001.zip › ijms-1186298-SI.pdf]

Evaluation of the PSMA-binding Ligand ^{212}Pb-NG001 in Multicellular Tumour Spheroid and Mouse Models of Prostate Cancer

Vilde Yuli Stenberg[1,2,3,*], Roy Hartvig Larsen[2], Li-Wei Ma[1], Qian Peng[4], Petras Juzenas[4], Øyvind Sverre Bruland[3,5] and Asta Juzeniene[1]

[1]Department of Radiation Biology, Institute for Cancer Research, The Norwegian Radium Hospital, Oslo University Hospital, 0379 Oslo, Norway
[2]Department of Research and Development, Nucligen AS, 0379 Oslo, Norway
[3]Institute for Clinical Medicine, University of Oslo, 0318 Oslo, Norway
[4]Department of Pathology, The Norwegian Radium Hospital, Oslo University Hospital, 0379 Oslo, Norway
[5]Department of Oncology, The Norwegian Radium Hospital, Oslo University Hospital, 0379 Oslo, Norway

*Corresponding author:
V. Stenberg, Oslo University Hospital, Oslo, Norway
Tel.: +47 901 284 34, e-mail address: vilde.stenberg@rr-research.no

SUPPLEMENTARY RESULTS

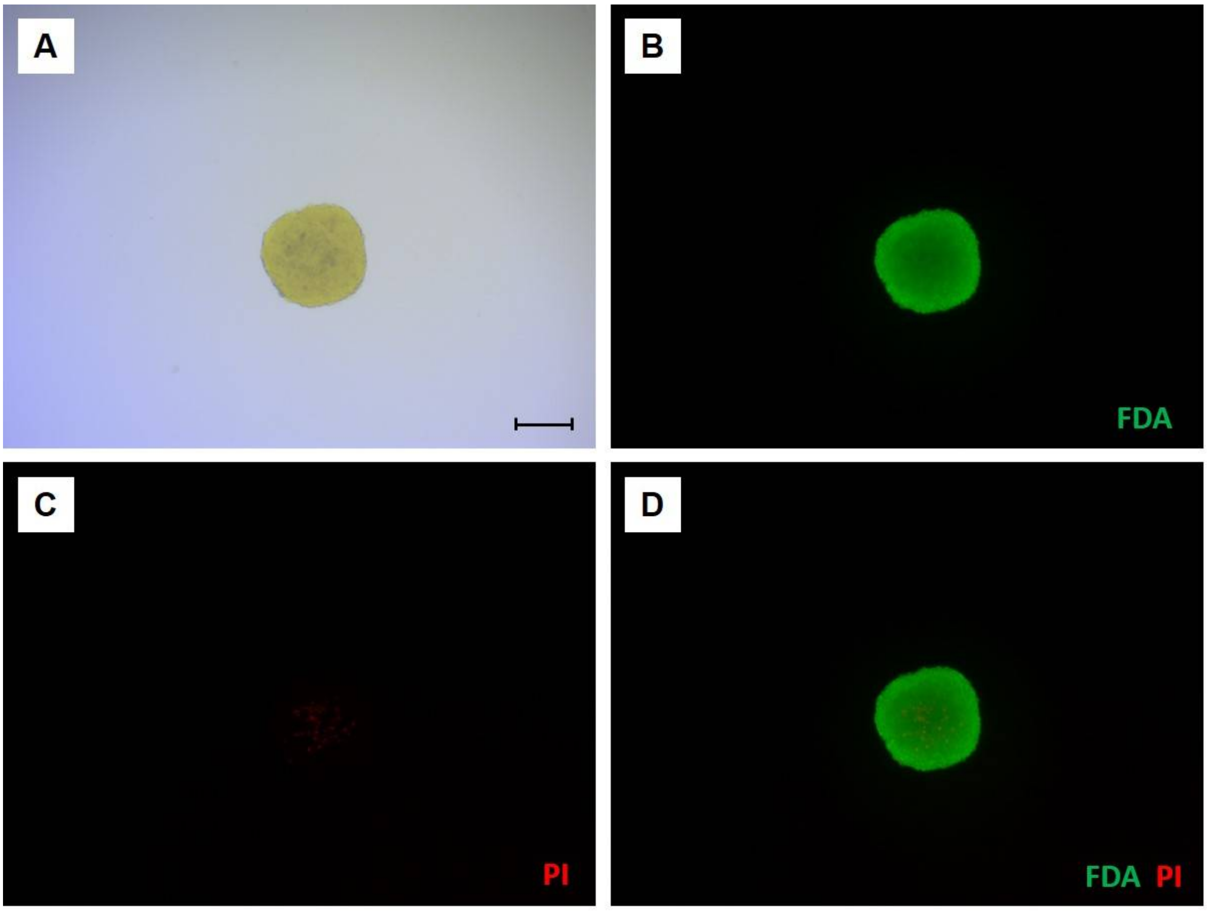

Figure S1. Microscope images (x4 magnification) of spheroids were taken at the day of treatment (day 0) using an inverted Axiovert 200M microscope (Zeiss, Germany) with AxioVision Rel. 4.8 software, scale bars are 200 µm. Representative bright field (A), FDA (B), PI (C) and merged FDA and PI (D) images are presented. The spheroids were incubated with fluorescein diacetate (FDA, 8 µg/ml, green channel for live cells) and propidium iodide (PI, 20 µg/ml, red channel for dead cells) for 5 minutes, and then washed with PBS containing Ca^{2+} and Mg^{2+} before imaging.

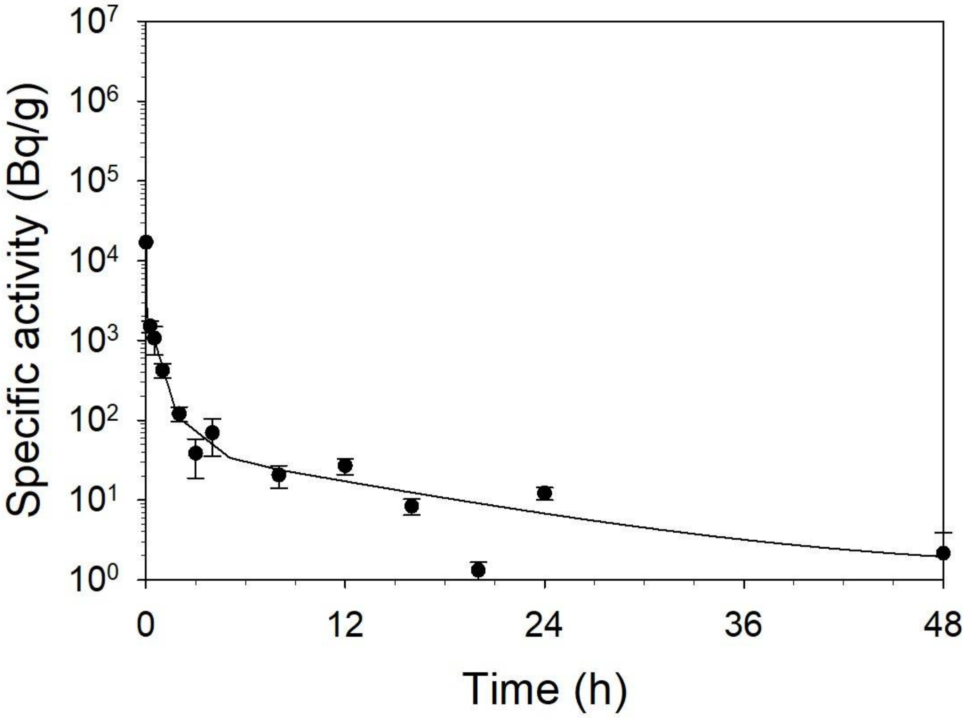

Figure S2. The clearance of ^{212}Pb-NG001 from blood of athymic nude mice bearing C4-2 xenografts, N=3-11.

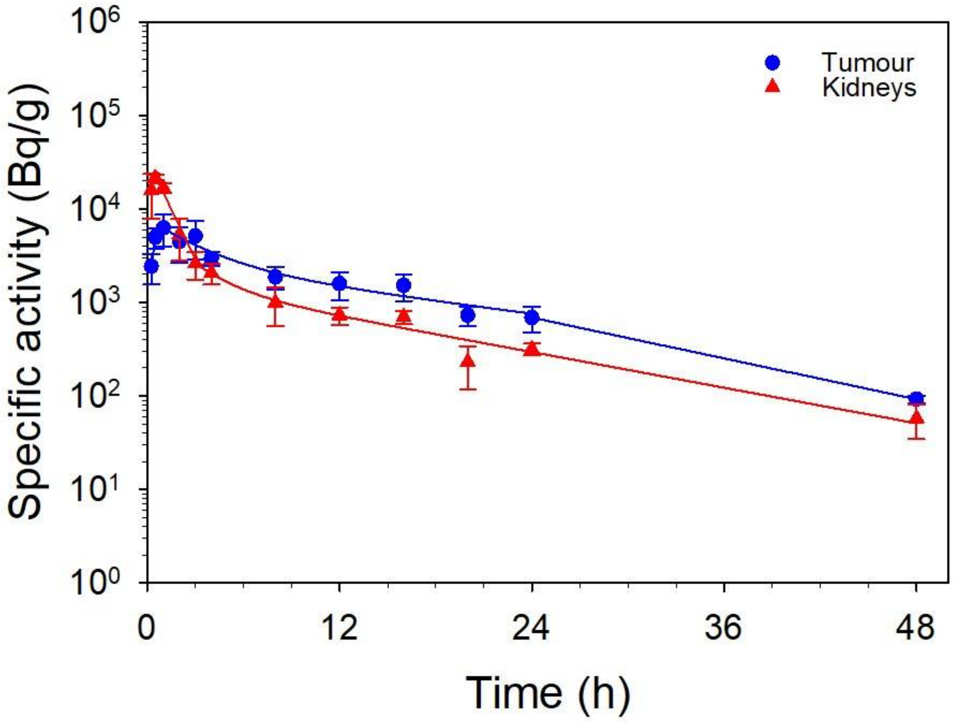

Figure S3. Uptake of ^{212}Pb-NG001 in tumour and kidneys of athymic nude mice bearing C4-2 xenografts, presented as Bq/g over time, N=3-11.

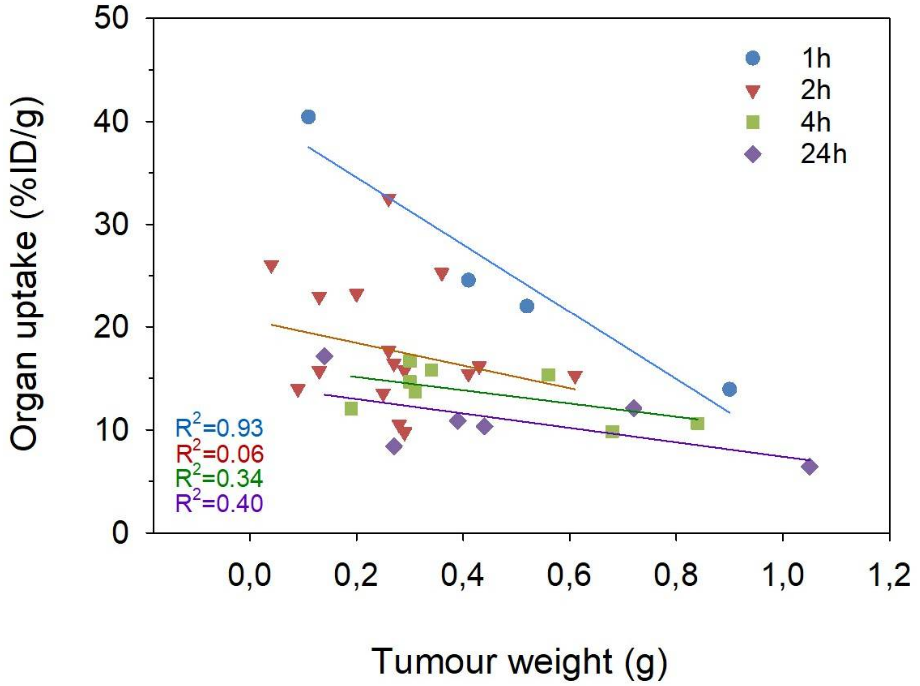

Figure S4. Tumour uptake (%ID/g) of ^{212}Pb-NG001 in C4-2 tumours of varying size at different time points post injection

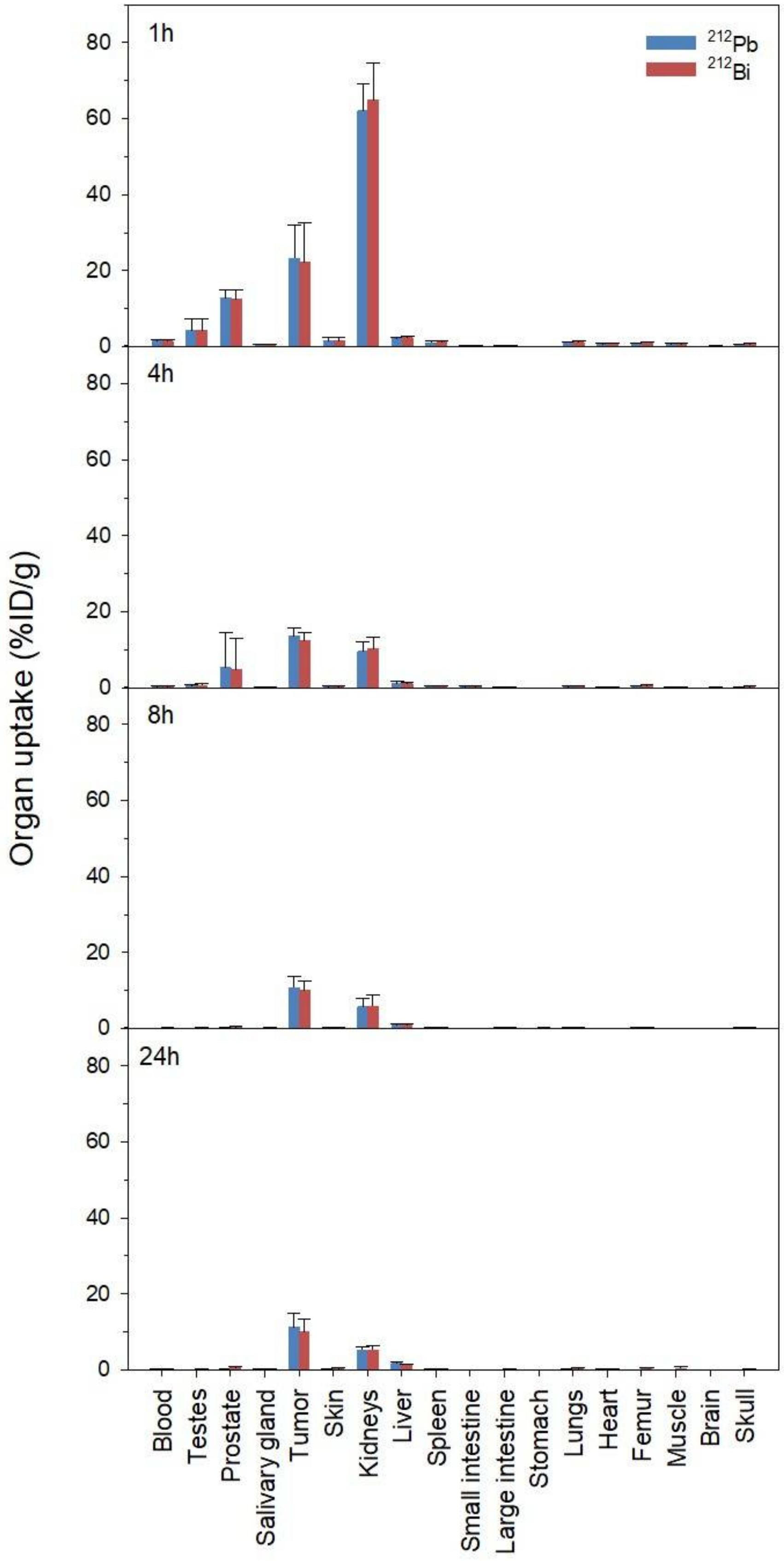

Figure S5. Uptake (%ID/g) ± SD of ^{212}Pb- and ^{212}Bi-labelled NG001 at different time points post injection in athymic mice bearing human prostate C4-2 xenografts, N=3-6.

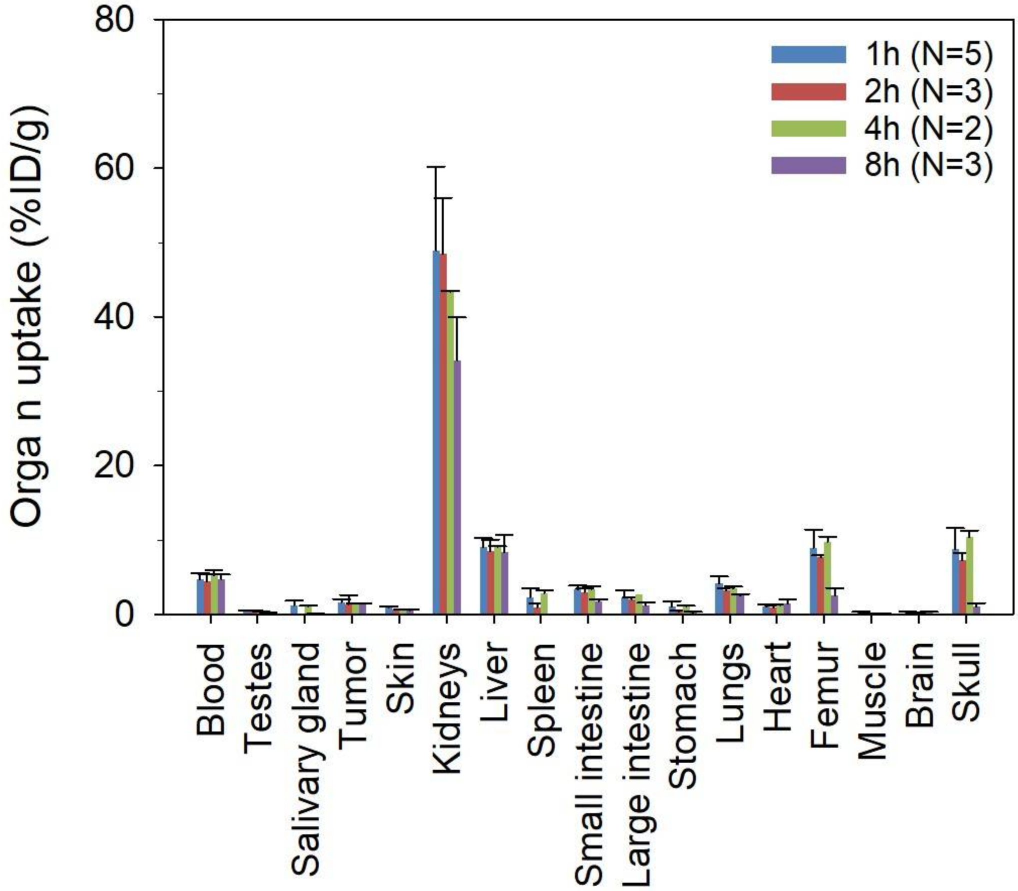

Figure S6. Decay-corrected percentage of injected activity per gram of tissue (%ID/g) ± SD of free ^{212}Pb in athymic nude mice (8-12 weeks in age) bearing human prostate C4-2 xenografts. N, number of mice per group.

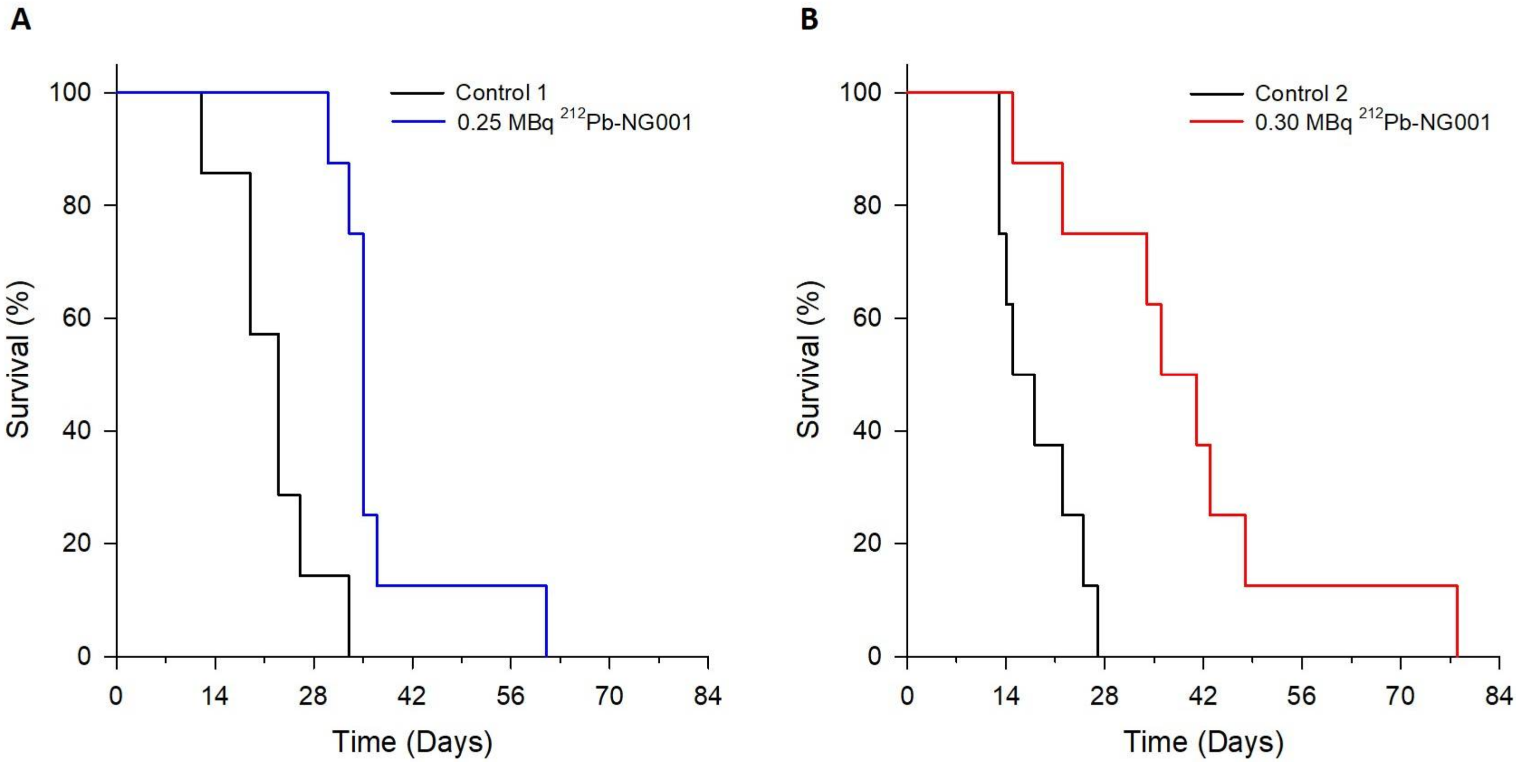

Figure S7. Survival of athymic nude mice bearing C4-2 tumours treated with saline (N=7-8), 0.25 MBq (N=8) or 0.30 MBq (N=8) of ^{212}Pb-NG001. Two independent experiments were performed (A and B). Survival were estimated by Kaplan-Meier survival analysis followed by the log-rank test by pairwise comparisons.

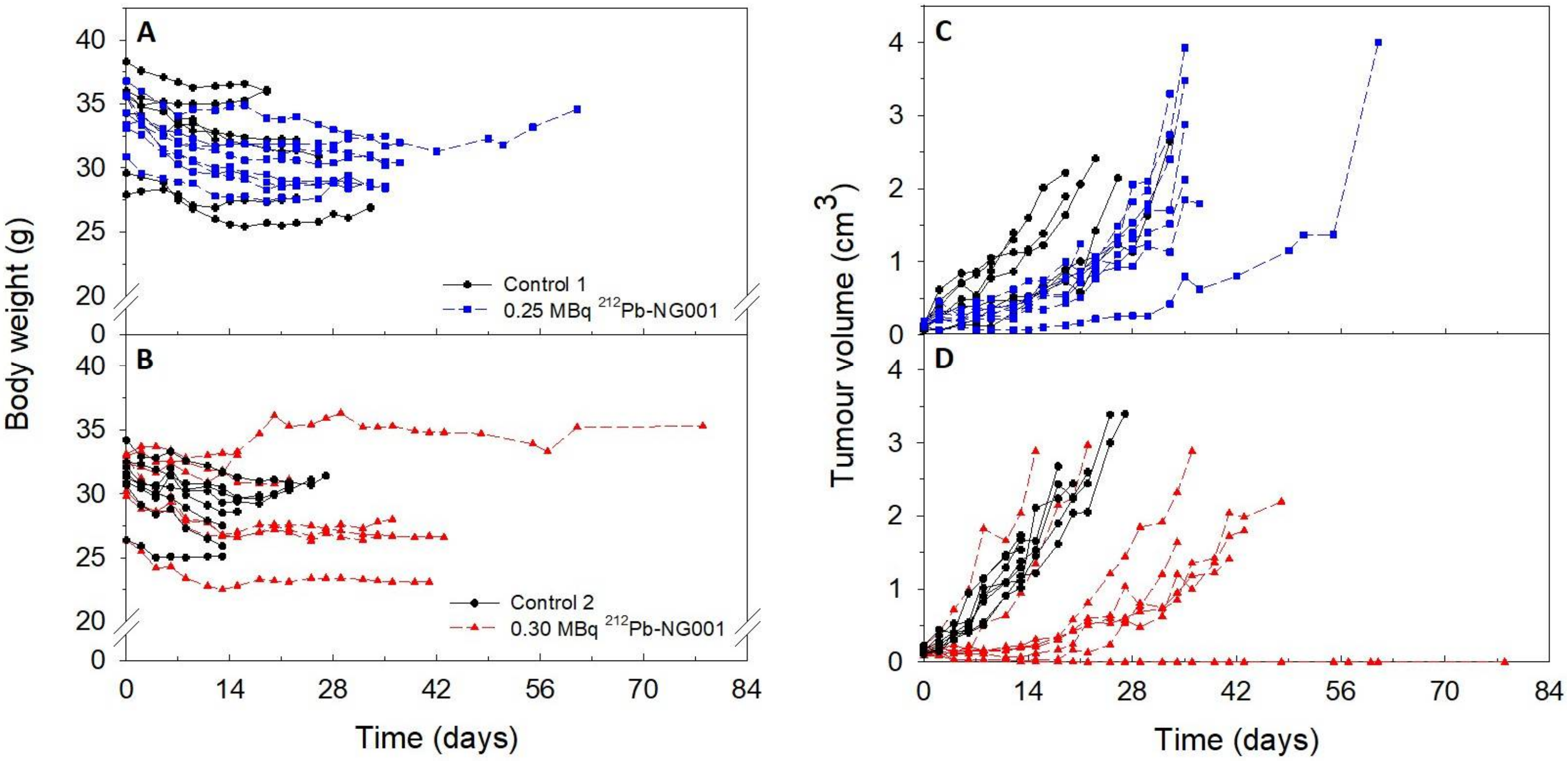

Figure S8. Changes in body weight (A, B) and tumour volume (C, D) of athymic nude mice bearing human prostate C4-2 cancer xenografts treated with saline (N=7-8), 0.25 MBq (N=8) or 0.30 MBq (N=8) of ^{212}Pb-NG001. Two independent experiments were performed (upper and lower), each line represents one mouse.

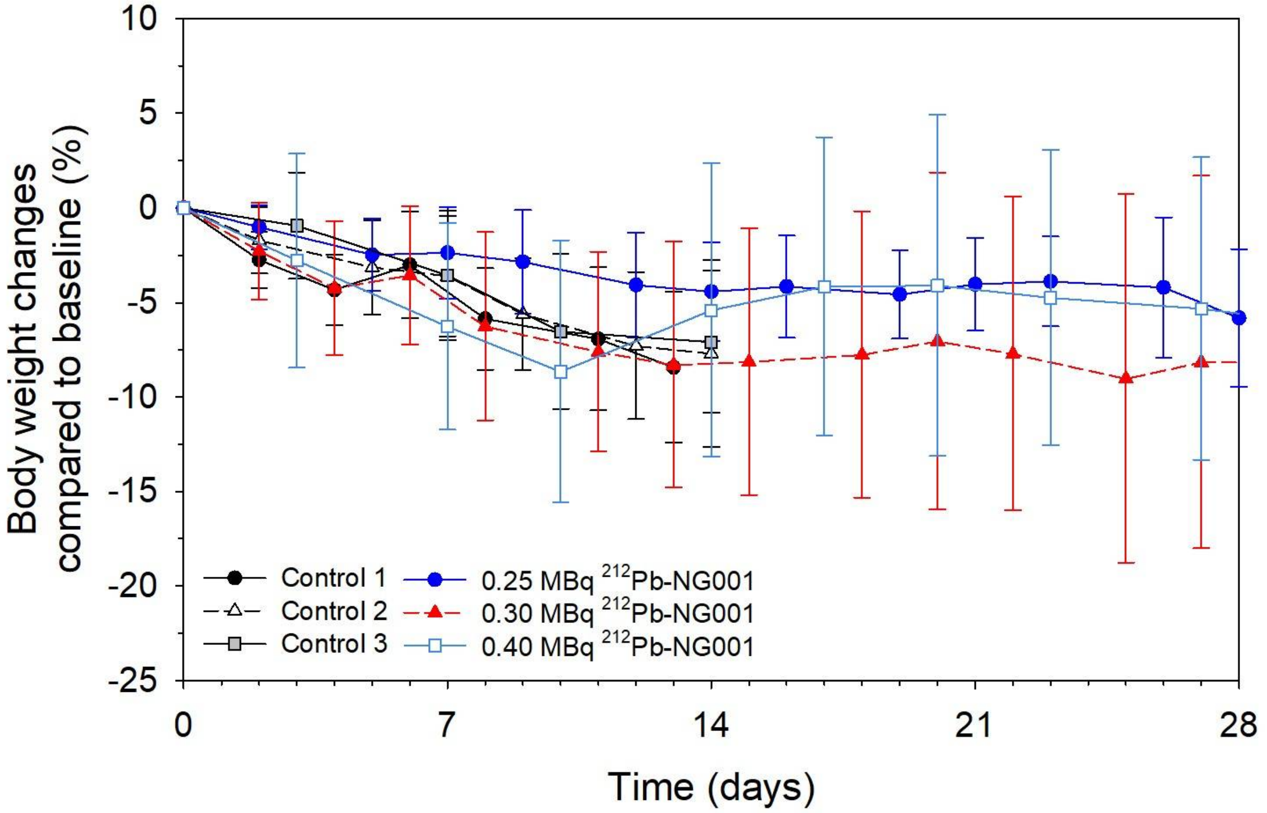

Figure S9. Body weight changes compared to baseline (%) of athymic nude mice bearing human prostate C4-2 cancer xenografts treated with saline (three independent experiments) or ^{212}Pb-NG001, N=6-8 mice per group.

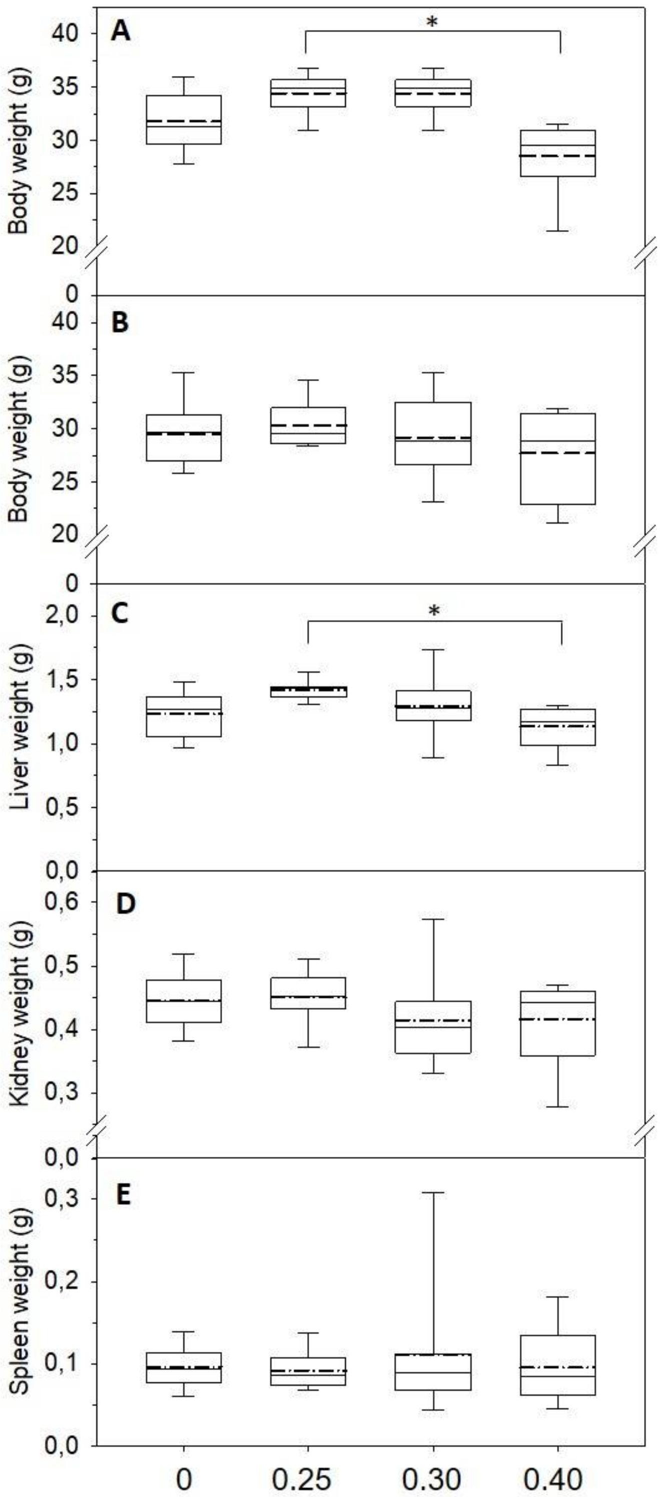

Figure S10. Body weight at the beginning of the study (A) and at the day of sacrifice (B) of athymic nude mice with human prostate C4-2 xenografts treated with saline (N=21), 0.25 MBq (N=8), 0.30 MBq (N=8) or 0.40 MBq (N=6) of ^{212}Pb-NG001. Weight of liver (C), kidneys (D) and spleen (E) at the day of sacrifice for the different treatment groups are presented. The control groups from the three independent experiments have been pooled to a single group. Bottom of the boxes represent the 25th percentile, dashed lines represent mean, solid lines represent medians, top of the boxes represent the 75th percentile and whiskers represent the 5th and 95th percentiles. $^{*}P<0.05$.

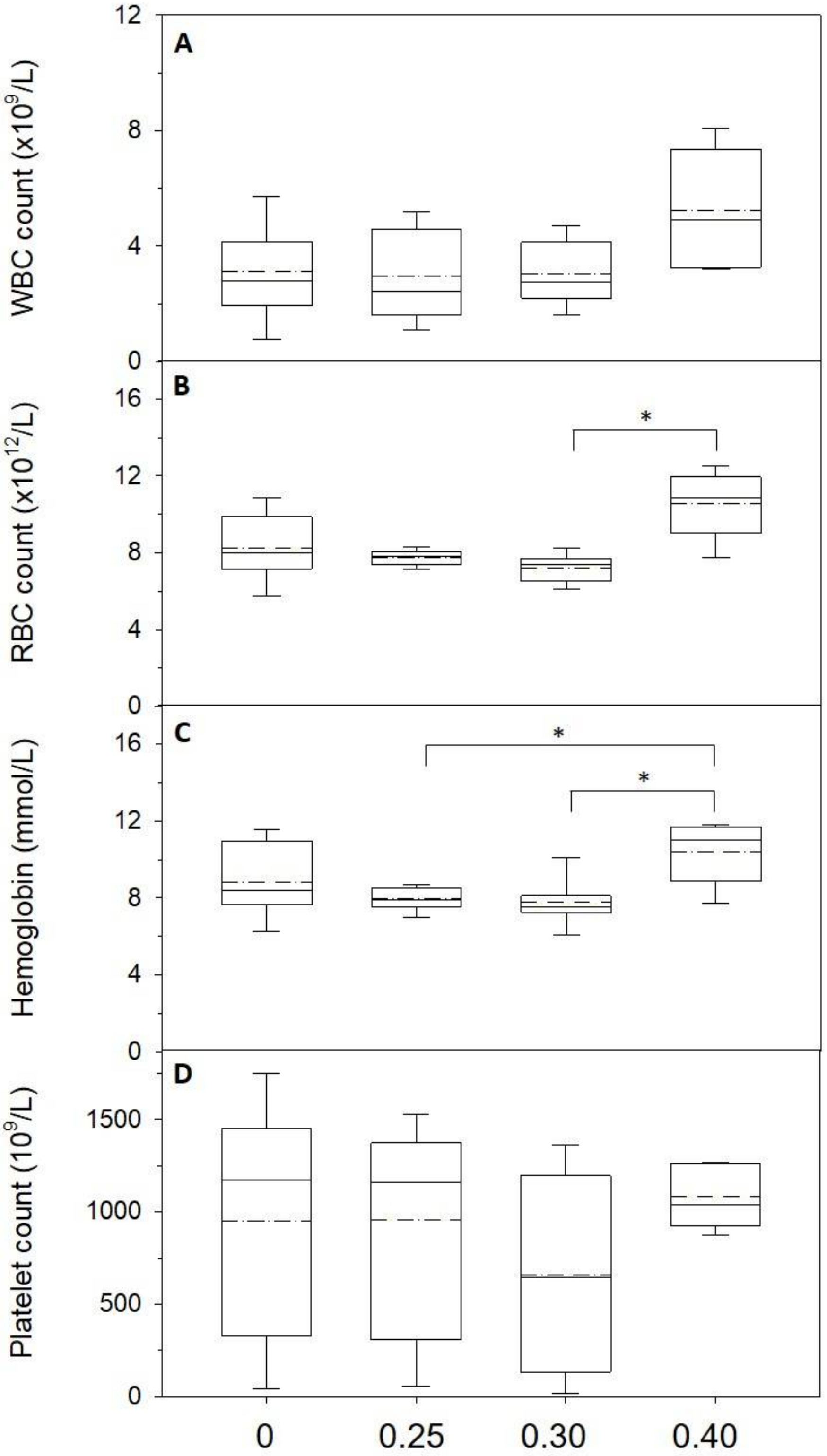

Figure S11. White blood cell (A), red blood cell (B), hemoglobin (C) and platelet (D) count of athymic nude mice with C4-2 xenografts treated with saline (N=21), 0.25 MBq (N=8), 0.30 MBq (N=6) or 0.40 MBq of ^{212}Pb-NG001. The control groups from the three independent experiments have been pooled to a single group.

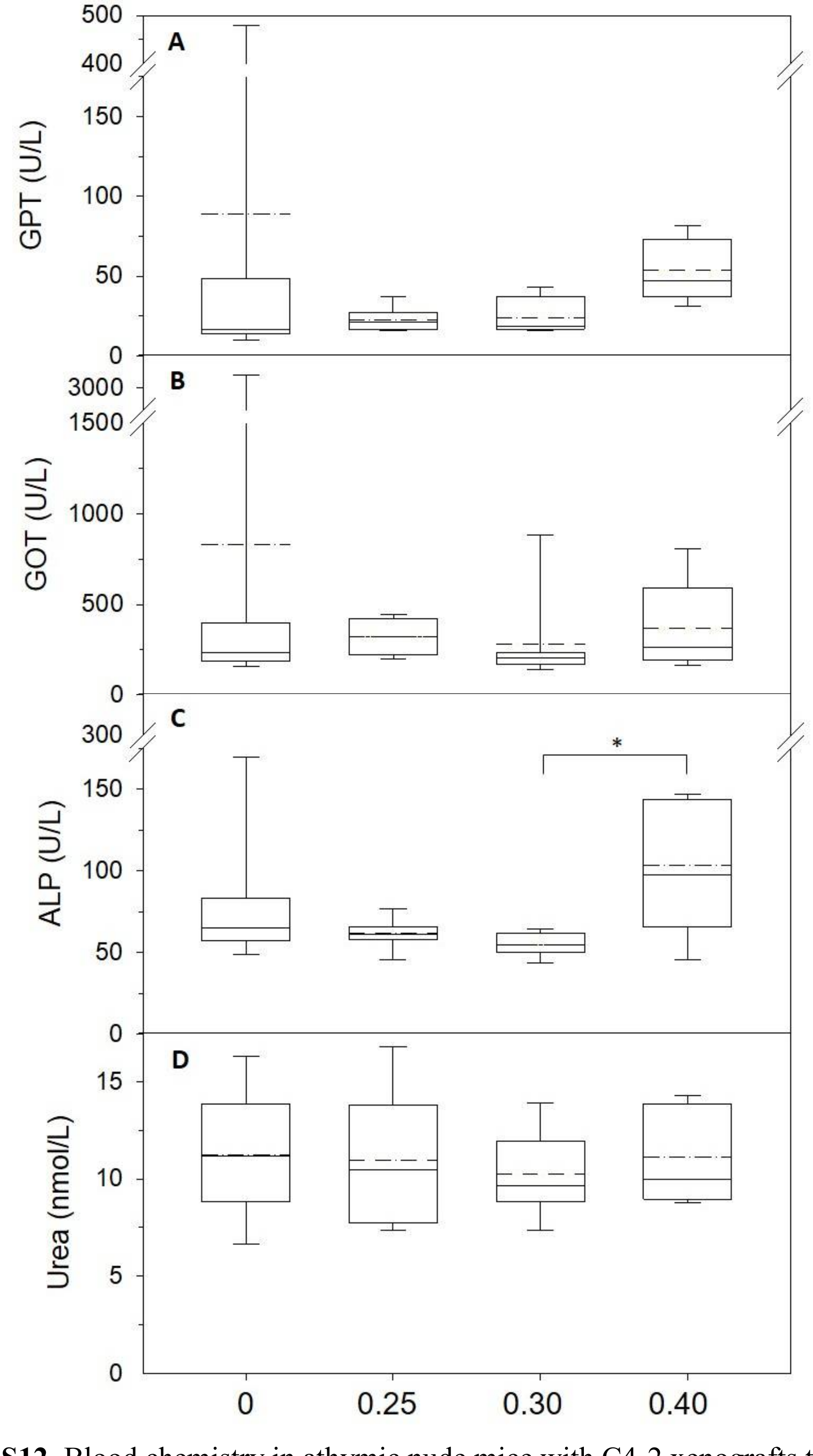

Figure S12. Blood chemistry in athymic nude mice with C4-2 xenografts treated with saline (N=21), 0.25 MBq (N=8), 0.30 MBq (N=8) or 0.40 MBq (N=6) of ^{212}Pb-NG001. Bilirubin <8.55 µmol/L and creatinine <44.2 µmol/L was measured for all mice, except for one control mouse with bilirubin of 31.3 µmol/L and one mouse treated with 0.30 MBq ^{212}Pb-NG001 with bilirubin of 8.76 µmol/L (results not shown). The control groups from the three independent experiments have been pooled to a single group. GOT, glutamic oxaloacetic transaminase; GPT, glutamic pyruvic transaminase; ALP, alkaline phosphatase.

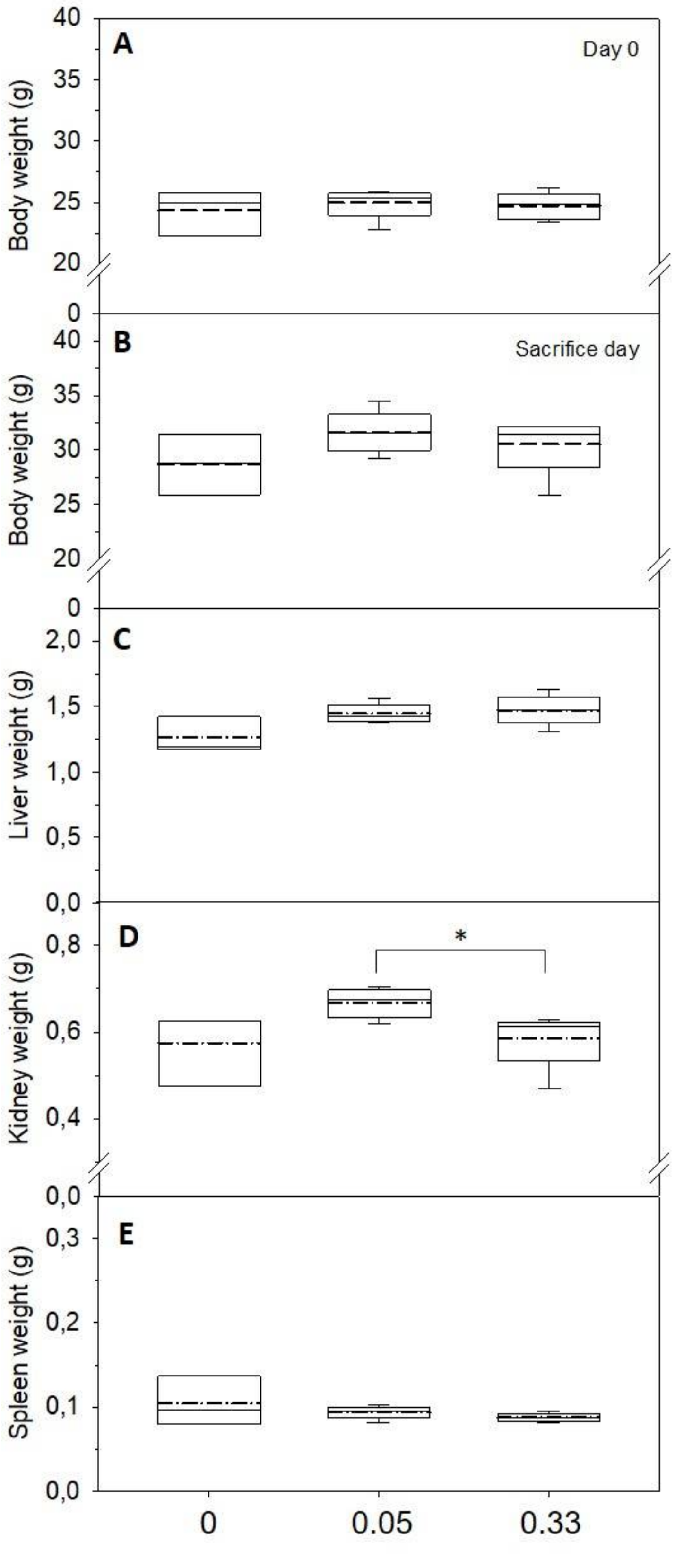

Figure S13. Body weight at the beginning of the study (A) and at the day of sacrifice (B) of BALB/c mice after administration with saline (N=3), 0.05 MBq (N=5) or 0.33 MBq (N=5) of ^{212}Pb-NG00. Weight of liver (C), kidneys (D) and spleen (E) at the day of sacrifice are presented.

Table S1. Therapeutic effect of various radiolabelled PSMA ligands in different subcutaneous animal models. The therapeutic index (TI) is calculated as the median survival of the treatment group divided by that of the control group. N/A, not available.

Radioligand	Mouse strain (cell line)	Injected activity (specific activity)	TI (P-value)	Reference
^{212}Pb-NG001	Hsd: Athymic Nude-Foxn1^{nu} (C4-2)	0.25 MBq (0.5 MBq/nmol)<br>0.30 MBq (1.1 MBq/nmol)<br>0.40 MBq (2.3 MBq/nmol)	1.5 (<0.001)/1.8 (0.002)*<br>2.3 (0.012)/2.5 (0.015)*<br>2.7 (<0.001)/2.5 (0.002)*	Current study
^{212}Pb-L2	NOD-SCID gamma (PC-3 PIP)	1.50 MBq (N/A)<br>3.70 MBq (N/A)	1.9 (0.003)**<br>3.0 (0.003)**	Banerjee et al., 2020 [24]
^{177}Lu-PSMA-617	SCID (C4-2)	15 MBq (84 MBq/nmol)	1.7 (<0.0001)	Lückerath et al., 2018 [58]
	NOD-SCID gamma (PC-3 PIP)	111 MBq (N/A)	3.9 (<0.0001)	Banerjee et al., 2019 [3]
	NOD rag gamma (LNCaP)	18.5 MBq (N/A)	4.1 (<0.05)	Kuo et al., 2018 [30]
	C57BL/6 (RM1-PGLS)	30 MBq (62 MBq/nmol)<br>60 MBq (62 MBq/nmol)<br>120 MBq (62 MBq/nmol)	1.4 (N/A)<br>1.9 (N/A)<br>1.9 (N/A)	Fendler et al., 2017 [36]
	Athymic nude BALB/c (PC-3 PIP)	2 MBq (2 MBq/nmol)<br>5 MBq (5 MBq/nmol)	1.1 (>0.05)<br>1.8 (<0.05)	Umbricht et al., 2018 [60]
	NOD-SCID gamma (C4-2)	30 MBq (84 MBq/nmol)	3.1 (≤0.001)	Stuparu et al., 2020 [59]
^{225}Ac-PSMA-617	NOD-SCID gamma (C4-2)	0.02 MBq (0.13 MBq/nmol)<br>0.04 MBq (0.13 MBq/nmol)<br>0.10 MBq (0.13 MBq/nmol)	3.6 (<0.014)<br>6.7 (<0.014)<br>4.2 (<0.014)	Stuparu et al., 2020 [59]
	Athymic nude mice (RM1-PGLS)	0.03 MBq (0.13 MBq/nmol)	1.1 (0.102)	Czernin et al., 2020 [61]

*TI was calculated from the median time for tumours to reach a volume of 1.5 cm^3 in the treatment groups compared to the control group. **TI was calculated as the median time to reach a 10-fold tumour increase for the treatment group divided by that of the control group.

REFERENCES

3. Banerjee, S. R.; Kumar, V.; Lisok, A.; Chen, J.; Minn, I.; Brummet, M.; Boinapally, S.; Cole, M.; Ngen, E.; Wharram, B.; Brayton, C.; Hobbs, R. F.; Pomper, M. G., (177)Lu-labeled low-molecular-weight agents for PSMA-targeted radiopharmaceutical therapy. *Eur. J. Nucl. Med. Mol. Imaging* **2019,** *46* (12), 2545-2557.

24. Banerjee, S. R.; Minn, I.; Kumar, V.; Josefsson, A.; Lisok, A.; Brummet, M.; Chen, J.; Kiess, A. P.; Baidoo, K.; Brayton, C.; Mease, R. C.; Brechbiel, M.; Sgouros, G.; Hobbs, R. F.; Pomper, M. G., Preclinical Evaluation of (203/212)Pb-Labeled Low-Molecular-Weight Compounds for Targeted Radiopharmaceutical Therapy of Prostate Cancer. *J. Nucl. Med.* **2020,** *61*, 80-88.

30. Kuo, H. T.; Merkens, H.; Zhang, Z.; Uribe, C. F.; Lau, J.; Zhang, C.; Colpo, N.; Lin, K. S.; Benard, F., Enhancing Treatment Efficacy of (177)Lu-PSMA-617 with the Conjugation of an Albumin-Binding Motif: Preclinical Dosimetry and Endoradiotherapy Studies. *Mol. Pharm.* **2018,** *15* (11), 5183-5191.

36. Fendler, W. P.; Stuparu, A. D.; Evans-Axelsson, S.; Luckerath, K.; Wei, L.; Kim, W.; Poddar, S.; Said, J.; Radu, C. G.; Eiber, M.; Czernin, J.; Slavik, R.; Herrmann, K., Establishing (177)Lu-PSMA-617 Radioligand Therapy in a Syngeneic Model of Murine Prostate Cancer. *J. Nucl. Med.* **2017,** *58* (11), 1786-1792.

58. Lückerath, K.; Wei, L.; Fendler, W. P.; Evans-Axelsson, S.; Stuparu, A. D.; Slavik, R.; Mona, C. E.; Calais, J.; Rettig, M.; Reiter, R. E.; Herrmann, K.; Radu, C. G.; Czernin, J.; Eiber, M., Preclinical evaluation of PSMA expression in response to androgen receptor blockade for theranostics in prostate cancer. *EJNMMI research* **2018,** *8* (1), 96-96.

59. Stuparu, A. D.; Capri, J. R.; Meyer, C.; Le, T. M.; Evans-Axelsson, S. L.; Current, K.; Lennox, M.; Mona, C. E.; Fendler, W. P.; Calais, J.; Eiber, M.; Dahlbom, M.; Czernin, J.; Radu, C. G.; Lückerath, K.; Slavik, R., Mechanisms of Resistance to Prostate-Specific Membrane Antigen-Targeted Radioligand Therapy in a Mouse Model of Prostate Cancer. *J. Nucl. Med.* **2020**, jnumed.120.256263.

60. Umbricht, C. A.; Benesova, M.; Schibli, R.; Muller, C., Preclinical Development of Novel PSMA-Targeting Radioligands: Modulation of Albumin-Binding Properties To Improve Prostate Cancer Therapy. *Mol. Pharm.* **2018,** *15* (6), 2297-2306.

61. Czernin, J.; Current, K.; Mona, C. E.; Nyiranshuti, L.; Hikmat, F.; Radu, C. G.; Lueckerath, K., Immune-Checkpoint Blockade Enhances ²²⁵Ac-PSMA617 Efficacy in a Mouse Model of Prostate Cancer. **2020**, jnumed.120.246041.
